# Experimental–Numerical Evaluation of Crack Growth in FeB/Fe_2_B Layers on AISI 316L Steel under Spherical Indentation

**DOI:** 10.1021/acsomega.5c12820

**Published:** 2026-06-10

**Authors:** Daybelis Fernández-Valdés, Adolfo López-Liévano, Alfonso Meneses-Amador, Dayvis Fernández-Valdés, Arturo Ocampo-Ramírez, Cristian Gómez-Rodríguez

**Affiliations:** † 27870 Universidad Veracruzana, Facultad de Ingeniería, Campus Coatzacoalcos, Av. Universidad Veracruzana km. 7.5, Col. Santa Isabel, Coatzacoalcos, Veracruz 96538, México; ‡ Instituto Politécnico Nacional, Grupo Ingeniería de Superficies, SEPI-ESIME Zacatenco, Av. Luis Enrique Erro S/N, Unidad Profesional Adolfo López Mateos, Alcaldía Gustavo A. Madero, C.P., Ciudad de México 07738, México

## Abstract

This work investigates crack evolution in AISI 316L steel borided by powder-pack process at 1223 K for 2 and 6 h. Microscopy, X-ray diffraction (XRD), and nanoindentation confirmed the formation of FeB/Fe_2_B composite layers whose hardness and Young’s modulus increased with treatment time. Spherical indentation tests using a 3 mm indenter produced circular cracks in both systems, with more pronounced damage in the 6 h layer due to higher elastic energy storage and stress concentration within the coating. Finite element modeling in ANSYS, using the SMART Crack technique, accurately reproduced the indentation imprint geometry and the stress fields governing the mechanical response under spherical indentation, showing good agreement with the experimentally observed surface imprint. The findings demonstrate that the thicker and stiffer layer formed after 6 h promotes deeper crack propagation toward the Fe_2_B/substrate interface, whereas the thinner 2 h layer confines damage mainly within the FeB phase with no evidence of interfacial failure. These results provide relevant insights for the design and performance prediction of brittle composite layers subjected to concentrated loading.

## Graphical abstract

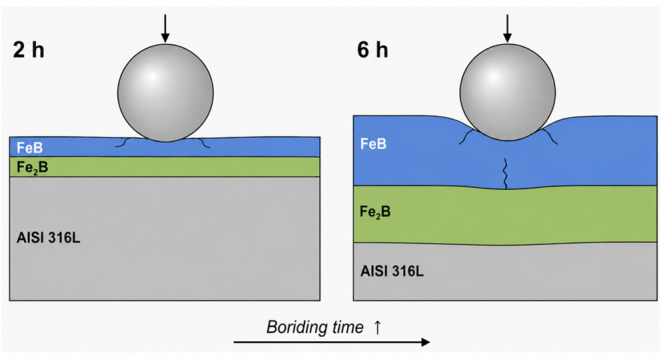

## 1 Introduction

Due to its versatility, corrosion resistance, and reasonable cost, AISI 316L stainless steel is widely used in various industries, including biomedical, petrochemical, automotive, and nuclear sectors.^1–3^ These applications justify ongoing efforts to improve its relatively low surface hardness, wear resistance, and corrosion resistance under demanding loading conditions through the use of thermochemical treatments as viable alternatives.^4–6^

One of the most cost-effective and efficient treatments is powder-packed boriding, which produces very hard coatings on steel and significantly enhances their surface performance.^7,8^ During this process, boron atoms diffuse into the substrate, forming hard boride layers that are inherently brittle and susceptible to both cohesive damage (crack formation) and adhesive failure (interfacial delamination). Understanding these failure mechanisms is essential to ensuring the industrial reliability of boride-coated components.

The most common approach to assess damage is through concentrated load conditions.^9–11^ In this context, spherical indentation is widely employed as an effective experimental technique to induce crack formation in coating-substrate systems and to evaluate their local mechanical response under controlled conditions.^11^ Numerous studies have demonstrated that, during spherical indentation, both the coating thickness and the mechanical properties of the material play a critical role in the fracture mechanisms, with surface circular cracks consistently identified as the predominant failure mode in hard coatings.^12–14^

In computational studies, Abdul-Baqui and Van der Giessen^15^ applied a cohesive zone model (CZM) to introduce cracks in thin films and interfaces, while Xiao et al.^9^ analyzed the relationship between coating crack formation and interfacial delamination, concluding that greater fracture strength in the film favors separation at the interface. These approaches, based on 2D models with predefined crack trajectories, have inherent limitations. To overcome these, Fakumasu et al.,^16^ Wang et al.,^17^ and Lu et al.^18^ used XFEM combined with CZM. However, 2D approaches cannot capture the stress intensity factor at the crack front and, therefore, its three-dimensional evolution. Therefore, this study employs the SMART Crack method in ANSYS, which evaluates crack extension and propagation without predefined trajectories. A 3D approach is adopted, enabling a more realistic representation of the evolution of elliptical crack fronts under spherical indentation conditions.

In this work, new experimental and numerical results are presented on the initiation and propagation of cracks in FeB/Fe_2_B layers formed on AISI 316L steel, using spherical indentation and three-dimensional modeling through the SMART Crack technique. A combined experimental-numerical approach is employed to relate the microstructure, crystalline phases, and mechanical properties of borided layers to critical damage loads, circular crack evolution, and the three-dimensional stress distribution. The results demonstrate that the layer thickness directly influences elastic energy accumulation and, consequently, the initiation of damage. This approach provides deeper insight into the failure mechanisms in borided systems and is highly relevant for components subjected to Hertzian contact.

## 2 Experimental Procedure

### 2.1 Powder-Pack Boriding and Mechanical Characterization

Cylindrical AISI 316L specimens, with a chemical composition of (wt %): 16.2% Cr; 10.5% Ni, 2.53% Mo, 0.76% Mn, 1.18% Si, 0.04% P and 0.03% C, were pack-borided at 1223 K for holding times of 2 and 6 h in a powder mixture of 10 wt % KBF_4_, 20 wt % B_4_C and 70 wt % SiC. The specimens, with dimensions of 10 mm in thickness and 30 mm in diameter, were embedded in sealed steel containers filled with the powder mixture to ensure a uniform boron activity during treatment. Prior to boriding, all surfaces were ground with SiC abrasive papers up to 2500 grit and polished to a mirror finish to guarantee reproducible starting conditions.

Cross sections were examined by optical microscopy (Olympus GX51), and phases were identified by X-ray diffraction (X’PERT PRO PANanalytical; Cu-Ka radiation at λ = 1.79; 2θ = 20°–80 °). The thicknesses of FeB and Fe_2_B layers were determined at several locations along the cross-section, and average values with standard deviations were reported to improve statistical reliability.

Through-thickness hardness (*H*) and Young’s modulus (*E*) profiles across the layer/substrate system were measured by instrumented nanoindentation using a Berkovich tip on a commercial NHT nanoindenter (CSM Instruments), in accordance with ISO 14577:2007. The mechanical properties of the layer were evaluated from the continuous recording of load and displacement obtained during the loading–unloading cycle. A maximum load of 100 mN was applied, with loading and unloading rates of 100 mN/min and a hold time of 10 s.

The hardness was determined from the applied load and the projected contact area (eq 1), while the elastic modulus (*E*) was calculated from the initial slope of the unloading curve (eq 2), according to the methodology proposed by Oliver and Pharr.^19^

Indentations were performed vertically from the surface toward the substrate, maintaining a minimal spacing of five times the contact radius to avoid plastic zone interaction. To ensure data reliability and repeatability, three separate indentation profiles were obtained for each condition.

1 $$H=\frac{P_{\max}}{24.5h_{c}^{2}}$$

2 $$E=\frac{1-{\left(v_{c}\right)}^{2}}{\frac{1}{E_{r}}-\frac{1-{\left(v_{i}\right)}^{2}}{E_{i}}}$$

where *P*_max_ represents the maximum applied indentation load, *h*_c_ indicates the contact depth, v_c_ and v_i_ refer to the Poisson ratio of the coating and indenter, respectively, *E*_r_ is the reduced elastic modulus and *E*_i_ denotes the elastic modulus of the indenter.

### 2.2 Spherical Indentation Testing

Spherical indentation tests were carried out on borided AISI 316L steel specimens treated for 2 and 6 h, using an alumina (Al_2_O_3_) spherical indenter of radius *R* = 3 mm. The experiments were conducted on an MTS Acumen electrodynamic testing system in load-controlled mode, ensuring proper fixation of the samples during the process. Monotonic loads ranging from 100 to 1000 N were applied in order to identify the load associated with the formation of circular cracks on the coating surface, considered as the first onset of cohesive damage under monotonic loading. After each test, the indenter was inspected for damage or deformation and replaced when necessary. Each condition was evaluated in triplicate at different surface locations, and the generated imprints were analyzed using an Olympus GX 51 optical microscope. The depth versus radial distance profiles of the indentation imprints in AISI 316L steel were obtained by optical profilometry through surface profile measurements.

## 3 Numerical Analysis

### 3.1 Static Analysis

Numerical simulations of the spherical indentation test were performed using the commercial software ANSYS 2025 R1. To validate the numerical model against the experiment (residual indentation depth *h* and contact radius *a*), a static analysis was performed for both systems using three load levels, 600, 650, and 700 N. Following the experimental setup, the base of the specimen was fixed, and a frictionless support was applied around the specimen to restrict only the surface-normal motion (Figure 1a). The protocol consisted of two steps: (I) load application and (II) removal of the spherical indenter by an upward displacement. The total thicknesses *t* of the boride layer, composed of the FeB and Fe_2_B phases, was obtained from experimental measurements and, given their brittle nature, these layers were modeled as isotropic elastic materials.

**Figure 1:**
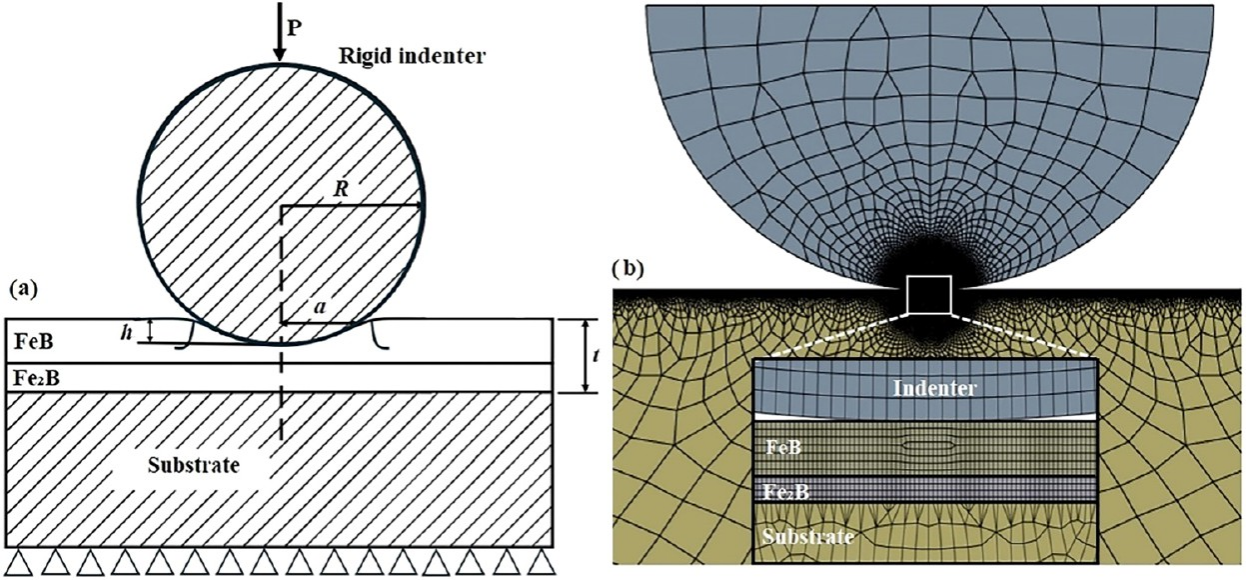
Schematic illustration of the indentation model: (a) geometry and boundary conditions, and (b) FEM mesh configuration.

Although the FeB and Fe_2_B layers exhibit gradients of mechanical properties across their thickness, a homogeneous representation with effective properties was deliberately adopted in this study. This type of idealization has been commonly employed in numerical studies of indentation and fracture in coating-substrate systems when the objective is to analyze the dominant mechanisms of stress redistribution and cohesive crack propagation, rather than to reproduce the complete microstructural gradient.^9,20–22^

In contrast, the AISI 316L substrate was defined as an elastoplastic material using a bilinear isotropic hardening model, characterized by its elastic modulus *E*, yield strength σ_y_ and tangent modulus *E*_T_, as summarized in Table 1. This material model was chosen to reproduce the overall elastic plastic recovery of the substrate under spherical indentation loading. To faithfully capture the stress and strain gradients, the mesh was refined in the indenter–specimen contact region (Figure 1b).

**Table 1:** Mechanical Properties Used In Numerical Analysis

condition	material	(E) [GPa]	Poisson’s (υ)	σ_*y*_ [MPa]	*E*_*T*_ [GPa][Bibr ref27]
2 h	FeB	353.3	0.23	-	-
	Fe_2_B	325	0.25	-	-
	AISI 316L	193	0.30	210	5
6 h	FeB	385.2	0.23	-	-
	Fe_2_B	350.7	0.25	-	-
	AISI 316L	193	0.30	210	5

In the numerical model, an initially stress-free state was assumed. The potential influence of residual stresses on the response obtained by nanoindentation has been widely discussed in the literature;^23–26^ however, in this work the mechanical properties used as input parameters were experimentally determined by nanoindentation across the coating-substrate system and represent an effective mechanical response of the overall system, which was directly employed in the numerical model.

A mesh-convergence study was performed by progressively refining the mesh from coarse to fine, confirming that variations in peak stresses within the contact region were negligible.

### 3.2 Crack Growth Analysis

Crack propagation was simulated using the SMART Crack method implemented in ANSYS, which automatically updates the crack-front shape and the surrounding mesh at each load increment. This method combines the evaluation of the J-integral and the stress intensity factors *K*_I_ obtained from multiple contours and incorporates an adaptive local remeshing strategy that enables fully three-dimensional crack propagation without the need to define a predefined crack path. The fracture toughness values of each phase were used as growth criteria, employing *K*_IC_ = 1.5 MPa √m for the FeB layer and *K*_IC_ = 3 MPa √m for the Fe_2_B layer, which corresponds to experimentally reported values for powder-boriding systems treated.^28^

In both systems, two initial cracks were introduced. The first corresponded to the superficial crack located in the FeB layer, positioned in the region where the maximum principal stress was concentrated and where, consistently, circular cracks were observed in the indentation tests. The second crack was placed at the Fe_2_B/substrate interface directly beneath the contact center, where the numerical analysis revealed a significant stress accumulation, which justifies evaluating fracture damage in this region. Crack growth was assessed under the same experimental loading conditions of 600, 650, and 700 N.

The cracks were modeled with a semielliptical geometry. In the FeB layer, minor and major radius values of 0.5–0.8 μm and 0.7–1.0 μm, respectively, were assigned. Similarly, in the Fe_2_B layer, radius values of 0.6–0.9 μm and 0.8–1.2 μm were used. The initial crack sizes were calibrated through a nongrowth step, ensuring that under the experimental load of each system, *K*_*I*_ increased until reaching the fracture toughness of the phase. This procedure ensured that the simulated crack propagation matched the experimentally observed crack initiation.

The initial geometry of the semielliptical crack was therefore explicitly calibrated to reproduce the crack initiation observed experimentally under spherical indentation. This calibration is not intended to represent the physical nucleation of microcracks, but rather to introduce a mechanically equivalent defect that allows for the analysis of crack propagation mechanisms under well-defined loading conditions.

Stress Intensity Factor *K*_I_ was selected as the criterion to evaluate crack growth and propagation in the system, as it was the dominant mode in the simulations. The contributions of *K*_II_ and *K*_III_ were practically negligible for both the superficial and interfacial cracks.

### 3.3 Calculation of Stress Intensity Factor *K*_I_

The Mode I stress intensity factor *K*_I_ used by SMART Crack is based on the classical fracture mechanics formulation^29^ implemented in ANSYS Mechanical. At each substep, the solver evaluates the *J*-integral using the domain integral method, which computes the strain energy density and displacement gradients around the crack front. Conventionally, the *J*-integral is expressed as

3 $$J=\int_{\Gamma }\left(Wn_{x}-\sigma_{\mathrm{ij}}n_{j}\frac{\partial u_{i}}{\partial x}\right)d\Gamma$$

where *W* is the strain energy density and Γ is the integration contour. In linear elasticity, the following energy relation is used

4 $$G=\frac{K_{I}^{2}}{E^{\prime }}$$

From which

5 $$K_{I}=\sqrt{JE^{\prime }}$$

where *E*′ = *E*/(1 – ν^2^) for plane strain conditions, or *E* for plane stress conditions. This relation is used internally by the CINT command, which evaluates the J-integral through the domain integral method and automatically converts its values into *K*_I_ for each contour around the crack front.^30^

To ensure the accuracy of *K*_I_ and the appropriate crack extension, six integration contours were evaluated around the crack front. The initial mesh, with an initial element size of 0.40 μm, exhibited a deviation of 5–7%, indicating a more open response. With minimum element sizes in the range of 0.25–0.15 μm, five of the six contours showed closer agreement, with differences below 2–3% (Figure 2). This convergence, together with the correlation between the location of the maximum principal stress, the radius of the experimental circular crack, and the initiation of crack growth under the experimental load, improves the crack-growth prediction of the models.

**Figure 2:**
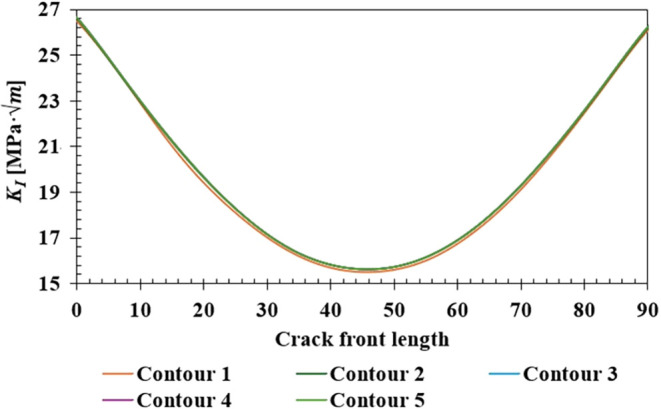
Convergence of (*K*_I_) stress intensity factor contours.

## 4 Results and Discussion

### 4.1 Powder-Pack Boriding Treatment

Figure 3(a and b) shows the cross-sectional micrographs of the borided AISI 316L steel. For the 2 and 6 h treatments, the FeB phase exhibits thicknesses of approximately 10.4 ± 0.3 μm and 36.3 ± 0.5 μm respectively, whereas the Fe_2_B phase reaches values of 6.2 ± 0.3 μm and 17.5 ± 0.5 μm. In both cases, a relatively flat morphology is observed, attributed to alloying elements such as chromium and nickel, which hinder the preferential diffusion of boron by reducing its active flux, leading to a flatter and less serrated boride layer morphology.^31,32^

**Figure 3:**
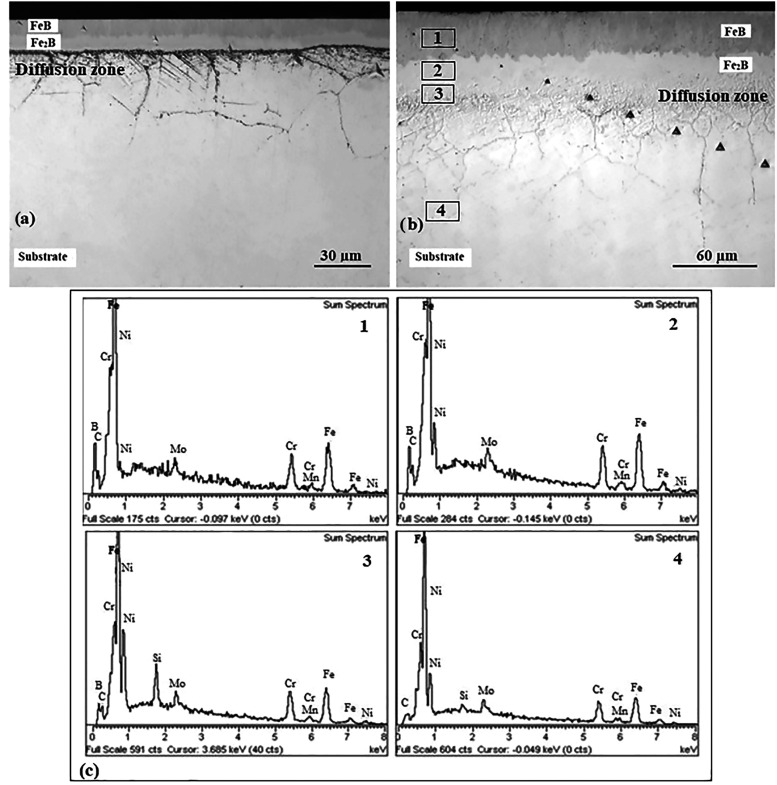
Cross-sectional micrographs of borided AISI 316L steel at (a) 2 h and (b) 6 h; (c) corresponding EDS spectra obtained at selected locations (points 1–4).

Figure 4 presents the X-ray diffraction patterns of borided AISI 316L steel for 2 and 6 h. In both conditions, the main detected phases correspond to the iron borides FeB and Fe_2_B, which are responsible for the formation of the compound layer. Additionally, secondary borides such as CrB and Cr_2_B are identified, associated with the chromium content of the alloy. This behavior is attributed to the high chromium concentration in the substrate, which allows its diffusion toward the boride layers, where it partially substitutes iron and reacts with boron, leading to the formation of chromium borides.^31^

**Figure 4:**
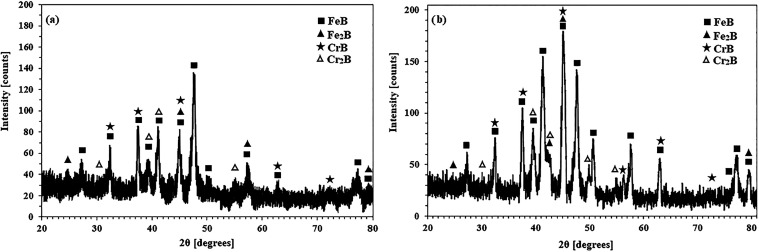
X-ray diffraction patterns of borided AISI 316L steel at 1223 K for (a) 2 h and (b) 6 h.

A clear difference between both conditions is observed in the intensity of the diffraction peaks. The 2 h sample exhibits lower intensity peaks, indicating a thinner and less developed boride layer. In contrast, the 6 h condition shows higher intensity peaks, consistent with a greater layer thickness due to the prolonged treatment.

In Figure 3c, the cross-sectional micrograph and the corresponding EDS spectra obtained at different locations (points 1–4) allow identification of the compositional variation across the FeB/Fe_2_B layers, the diffusion zone, and the substrate. The EDS results indicate the presence of elements such as Fe, Cr, Ni, and Mo in all analyzed regions; however, variations in signal intensity are observed along the cross-section, suggesting the presence of a compositional gradient throughout the analyzed section.

The hardness (*H*) and elastic modulus (*E*) profiles for both conditions are presented in Figure 5(a and b). The measurements were performed on cross sections, covering the region from the surface of the layer down to the substrate to capture the full variation of properties across the coating–substrate system. The condition with the thicker layer exhibited an increase in surface mechanical properties, reaching a maximum hardness of approximately 22 GPa, whereas the thinner layer displayed a value of about 20 GPa. A similar trend was observed in the elastic modulus, with maximum values of around 354 GPa for 2 h and 386 GPa for 6 h. Furthermore, no abrupt change in mechanical properties is observed across the system, which can be attributed to the diffusion-controlled nature of the boriding process. The formation of borided layers involves a gradual transition in composition and microstructure from the compound layer to the diffusion zone and the substrate, resulting in a continuous variation of mechanical properties. Prolonged treatment durations lead to increased values of *H* and *E*, which are linked to enhanced diffusion and boron enrichment at the surface. These effects contribute to greater mechanical resistance and promote the gradual expansion of the transition zone toward the substrate. The transition zone corresponds to the region between the boride layer and the substrate, as shown in Figure 3b, where the mechanical properties increase gradually from the substrate values toward those of the boride layer. Based on the combined analysis of the layer thickness and the hardness and elastic modulus profiles, it is possible to estimate the extent of this region. Under the 2 h condition, the transition zone extends up to approximately 40 μm, whereas under the 6 h condition, the longer exposure time leads to an extension of this zone up to approximately 100 μm

**Figure 5:**
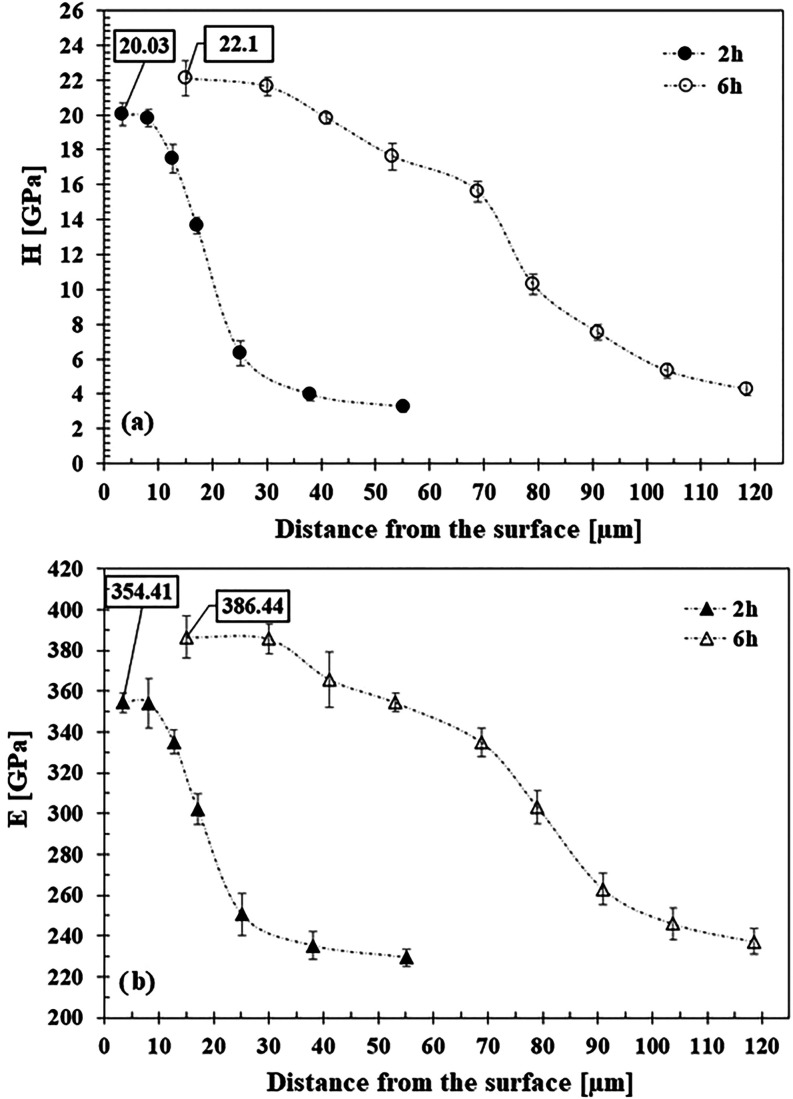
Hardness and Young’s modulus profiles obtained for borided AISI 316L steel.

### 4.2 Monotonic Indentation Test

Figure 6 shows the evolution of surface damage under monotonically increasing loads of 600, 650, and 700 N for the 2 h (a) and 6 h (b) treatment conditions. In the system with the thinner layer (2 h), no visible damage is observed at 600 or 650 N, whereas at 700 N fine circular cracks appear at the edge of the indentation impression, indicating the onset of cohesive damage. In contrast, in the 6 h system, which has thicker layers, circular cracks are already visible at 600 N and gradually intensify with increasing load, revealing a more brittle behavior.

**Figure 6:**
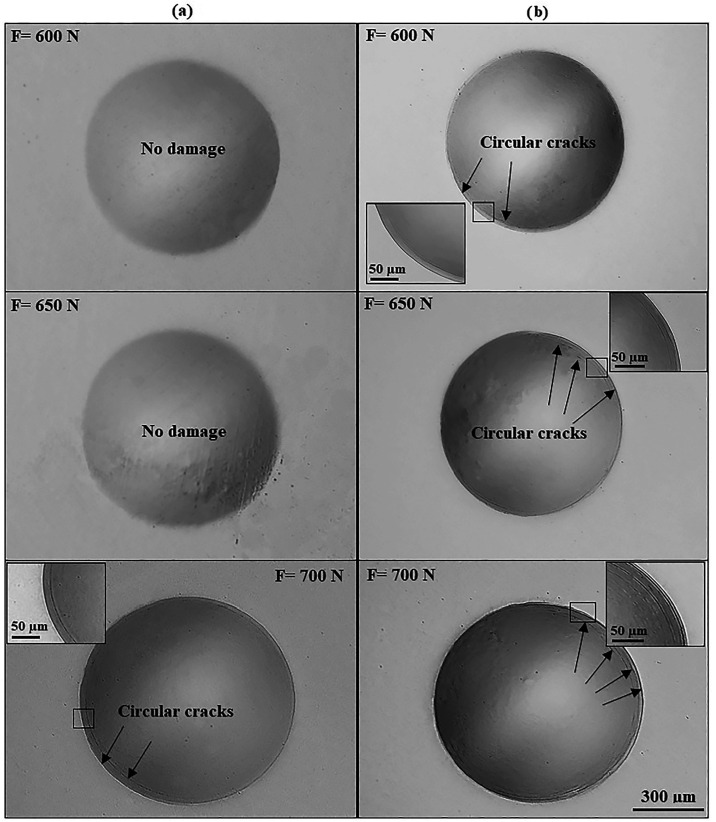
Evolution of cohesive damage under monotonic loading in powder-borided AISI 316L steel: (a) 2 h and (b) 6 h.

These results indicate that the 2 h system displays greater resistance to the onset of damage, as it preserves its surface integrity under higher applied loads before fracture initiates. At 700 N, the dominant fracture mode in both conditions corresponds to the formation of circular cracks; however, their characteristics differ markedly with boriding time. In the 6 h condition, the cracks are more numerous and more severe, reflecting a more pronounced degree of damage, while in the 2 h condition they appear in smaller number and with lower definition, indicating a tougher response and a higher resistance to damage propagation.

This contrast is attributed to the fact that longer treatment times produce a harder and stiffer boride layer with a lower deformation capacity, which promotes stress concentration on the surface and leads to earlier fracture. Similar behavior has been widely reported in coating-substrate systems, where harder and stiffer coatings tend to accumulate higher stresses and develop more severe cracking or delamination under concentrated loads.^15,18^

### 4.3 Finite Element Analysis

#### 4.3.1 Stress Analysis

Before analyzing stress distribution and crack growth, it was necessary to validate the numerical model implemented in ANSYS. For this purpose, the indentation profiles obtained experimentally were compared with those generated by the FEM simulation under a 700 N load. Figure 7 shows a good correlation between both approaches, supporting the validity of the developed numerical model. For the 2 h condition, the relative difference between the experimental and numerical profiles was approximately 6%, whereas for the 6 h condition it decreased to about 5.2%, confirming the capability of the model to accurately reproduce the indentation geometry.

**Figure 7:**
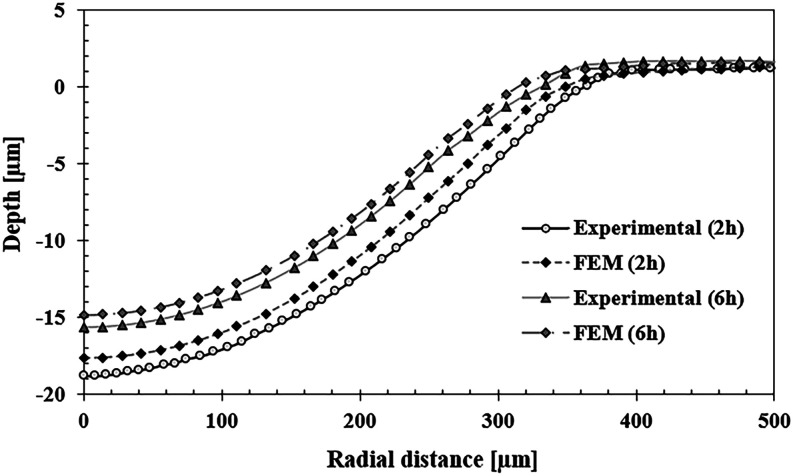
Experimental and numerical residual indentation depth profiles of powder-borided AISI 316L steel under a 700 N load.

Figure 8 presents the distribution of maximum principal stresses (MPa) obtained under monotonic spherical indentation in powder-borided AISI 316L steel treated for 2 and 6 h, showing a treatment-time-dependent mechanical response. In the 2 h condition, the smaller layer thickness limits the inward propagation of tensile stresses, confining them to the periphery of the indentation imprint as a consequence of the flexural response of the boride layer induced by plastic deformation of the substrate, which results in localized and less extensive damage. In contrast, in the 6 h condition, the thicker layer leads to a greater accumulation of elastic energy, which promotes the propagation of tensile stresses not only around the contact periphery but also toward the Fe_2_B/substrate interface. This effect increases the likelihood of circular crack formation and raises the possibility of interfacial delamination under high loads, reflecting a more severe damage response than in the 2 h condition. Consistently, several studies have reported that increasing the thickness of hard layers intensifies stress concentration and accelerates the propagation of damage toward the interface.^13,33,34^

**Figure 8:**
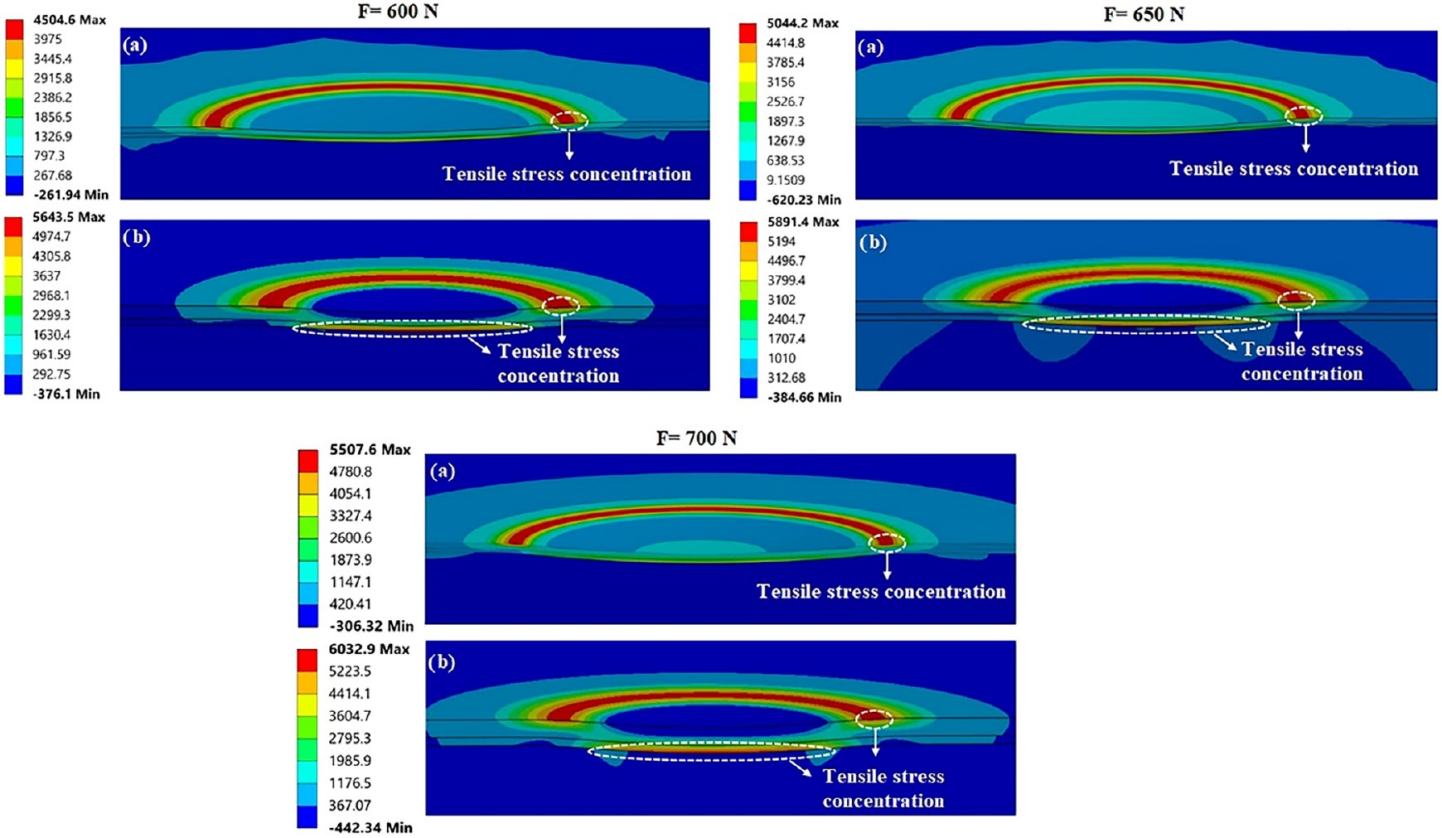
Distribution of maximum principal stresses (MPa) under spherical indentation of borided AISI 316L steel for (a) 2 h and (b) 6 h.

Figure 9 shows the evolution of the maximum principal stresses along the axis of symmetry during the loading and unloading stages for applied loads of 600, 650, and 700 N. During the loading stage, the highest stresses were located at the surface, reaching approximately 5236, 5545, and 6250 MPa for the 2 h condition, and 5752, 6145, and 6532 MPa for the 6 h condition, respectively. In the unloading stage, the stress decreases slightly due to the plastic relaxation of the substrate. In this phase, for the 6 h condition, the maximum principal stress, which during loading was located at the surface (peripheral contact zone), progressively shifts toward the Fe_2_B/substrate interface as unloading progresses. This phenomenon indicates a transfer of the stress field toward the interfacial region and confirms the presence of two critical damage zones in the system: a surface zone and an interface one, associated with layer bending and relaxation.

**Figure 9:**
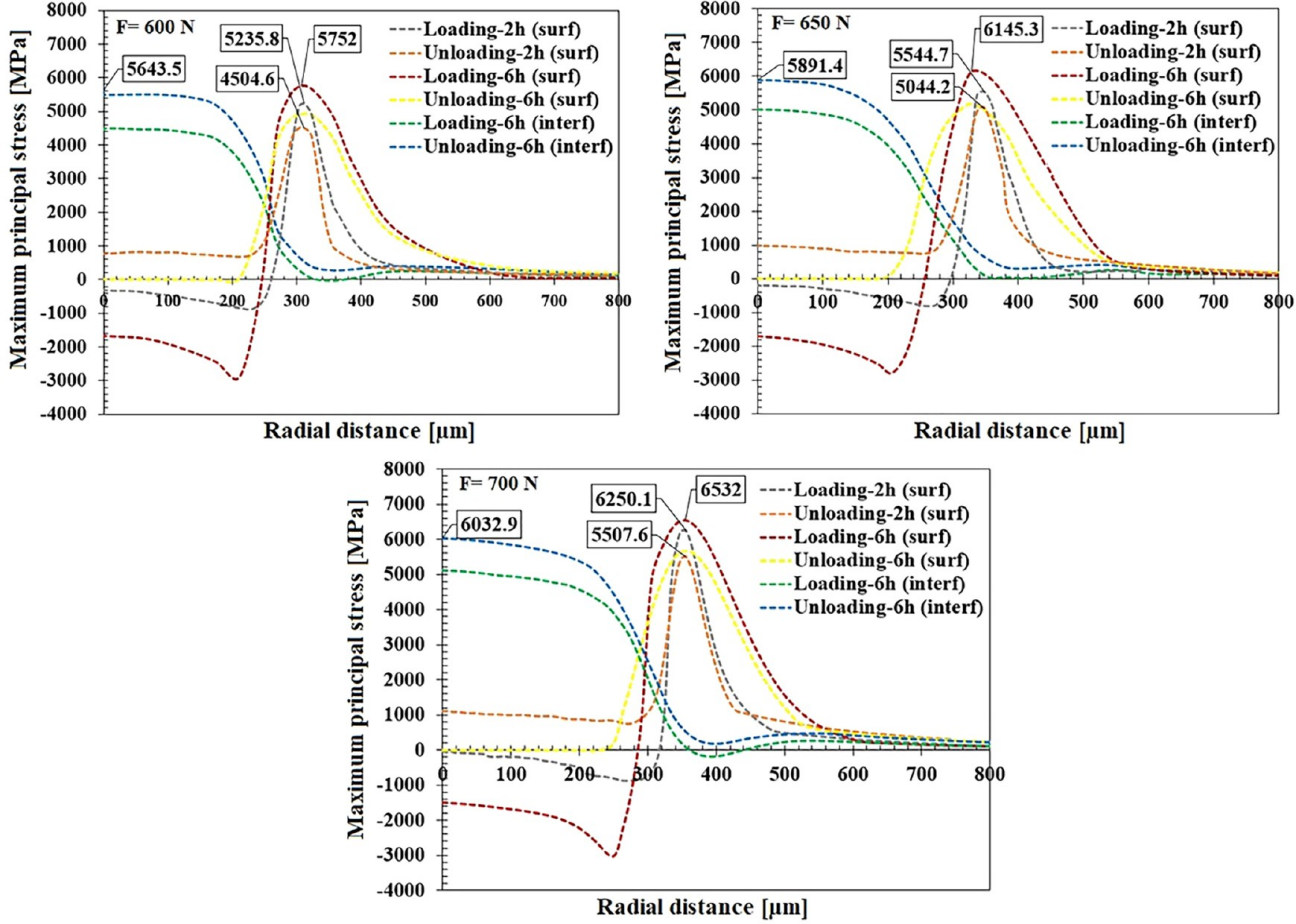
Evolution of maximum principal stresses (MPa) during the loading and unloading stages.

Figure 10 shows the distribution of shear stresses under spherical indentation during the loading stage for the 2 and 6 h treatment conditions at 600, 650, and 700 N. In both systems, the highest stress concentrations are located within the FeB layer and extended slightly toward the FeB/Fe_2_B interface, where the property gradient between both layers generates a discontinuity that favors stress accumulation. As the applied load increases, the shear stresses increase in both magnitude and spatial extent, reaching approximately 1102, 1260, and 1552 MPa for the 2 h condition, and 1561, 1970, and 2393 MPa for the 6 h condition, respectively. The greater layer thickness in the 6 h treatment promotes a broader distribution and higher stress concentrations, whereas the thinner 2 h layer maintains a more localized and stable stress field.

**Figure 10:**
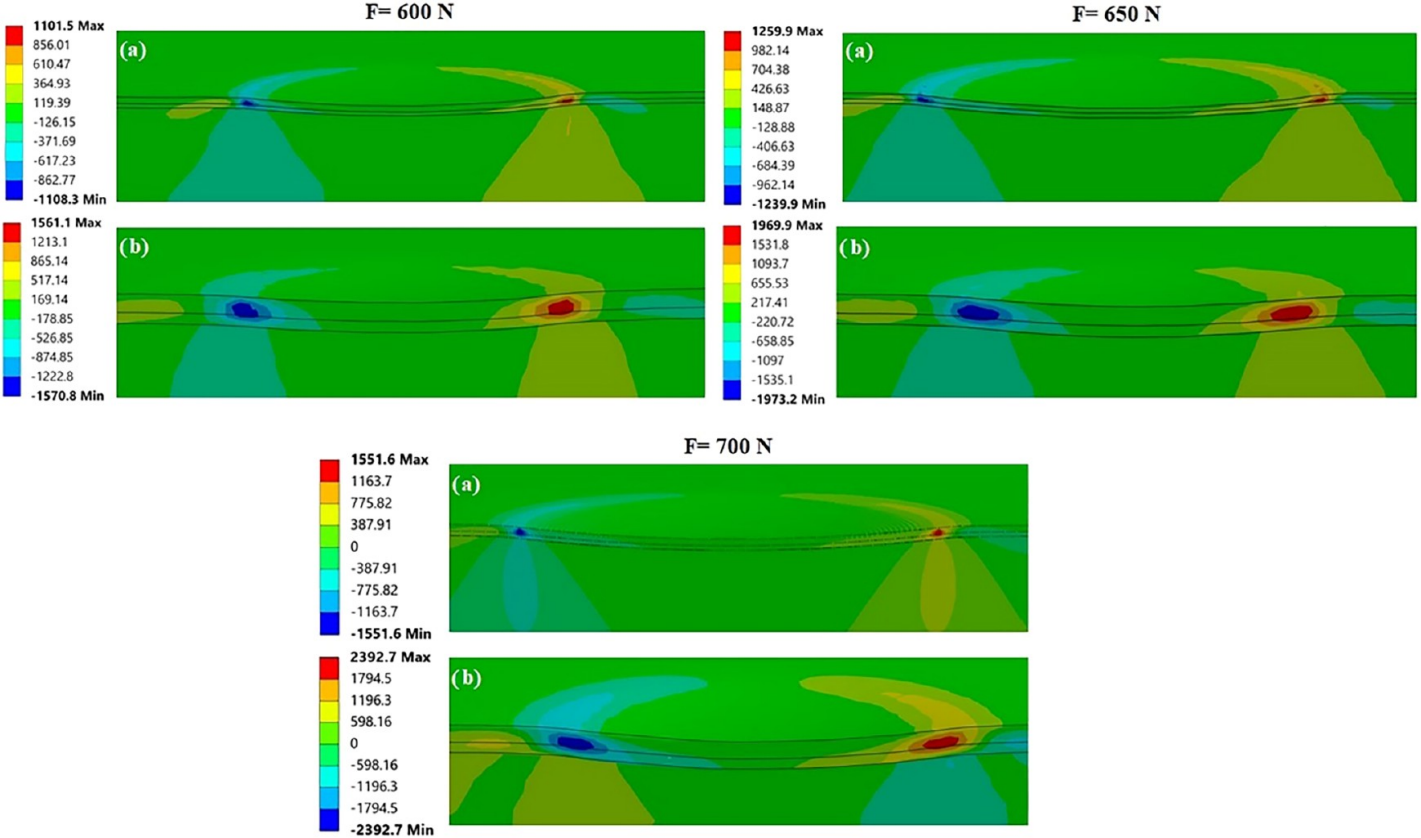
Shear stress distribution under spherical indentation of borided AISI 316L steel for (a) 2 h and (b) 6 h.

Figure 11 shows the evolution of shear stress along the FeB/Fe_2_B interface, obtained from the axis of symmetry, comparing the loading and unloading stages. During unloading, the stress decreases due to substrate relaxation, which leads to a redistribution of the stress field throughout the interface. In the 6 h condition, elevated concentrations and wider curves persist, indicating that the greater layer thickness not only increases the magnitude of the shear stresses but also their spatial extent along the interface, thereby increasing susceptibility to interfacial damage. In contrast, the 2 h condition exhibits more confined curves and greater interfacial stability under increasing load

**Figure 11:**
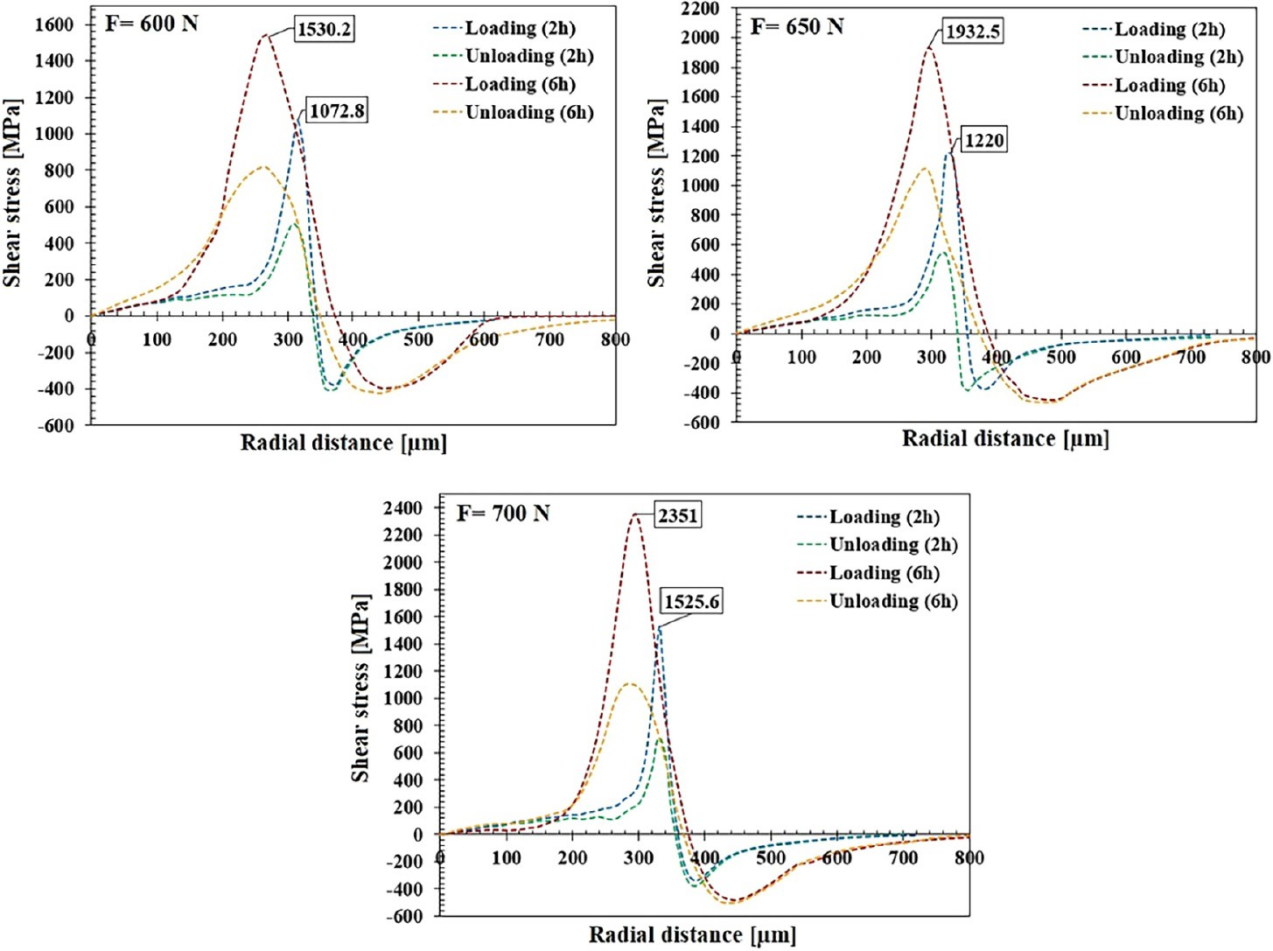
Shear stress distribution along the FeB/Fe_2_B interface during the loading and unloading stages.

### 4.4 Crack Growth Analysis

In this section, crack propagation is analyzed using the SMART Crack approach from a comparative perspective, focusing on the identification of dominant mechanical trends. The experimental support of the numerical model in this work is based on global contact parameters, namely the residual indentation depth (*h*) and the contact radius (*a*), which are commonly used to characterize the global mechanical response under Hertzian contact.^35^ The experimental observations are limited to top-view images, where circular surface cracks are consistently identified. Accordingly, the SMART Crack simulations are employed to establish relative comparisons between the 2 and 6 h boriding conditions and to provide mechanistic insight into the mechanical evolution of damage under indentation.

In Figure 12a, it shows that crack extension increases as the indentation load rises. The interfacial cracks grew upward from the Fe_2_B/substrate interface, reaching lengths of 9.06, 11.4, and 14.3 μm under 600, 650, and 700 N, respectively. Meanwhile, the cracks in the FeB layer propagated from the surface toward the interior of the layer. In the 6 h condition, their extensions were 10.5 μm under 600 N, 13.2 μm under 650 N, and reached a maximum of 17.7 μm under 700 N (Figures 13, 14, and 15). In contrast, the 2 h condition exhibited only superficial crack growth in FeB at 700 N, with an extension of 5.4 μm (Figure 16) and no interfacial crack growth was observed at any of the applied loads. This is because, in the thinner and less stiff layers, the stress concentrations are insufficient to exceed the fracture toughness of the Fe_2_B phase and therefore crack propagation does not initiate.

**Figure 12:**
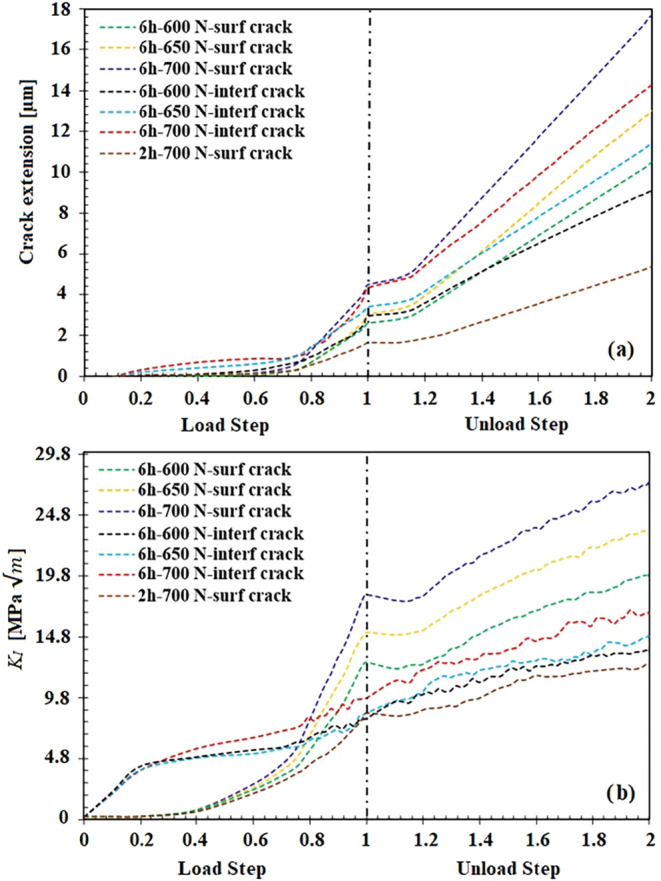
Crack extension (a) and *K*_I_ evolution (b) during the loading–unloading cycle at 700 N in the 2 and 6 h systems.

**Figure 13:**
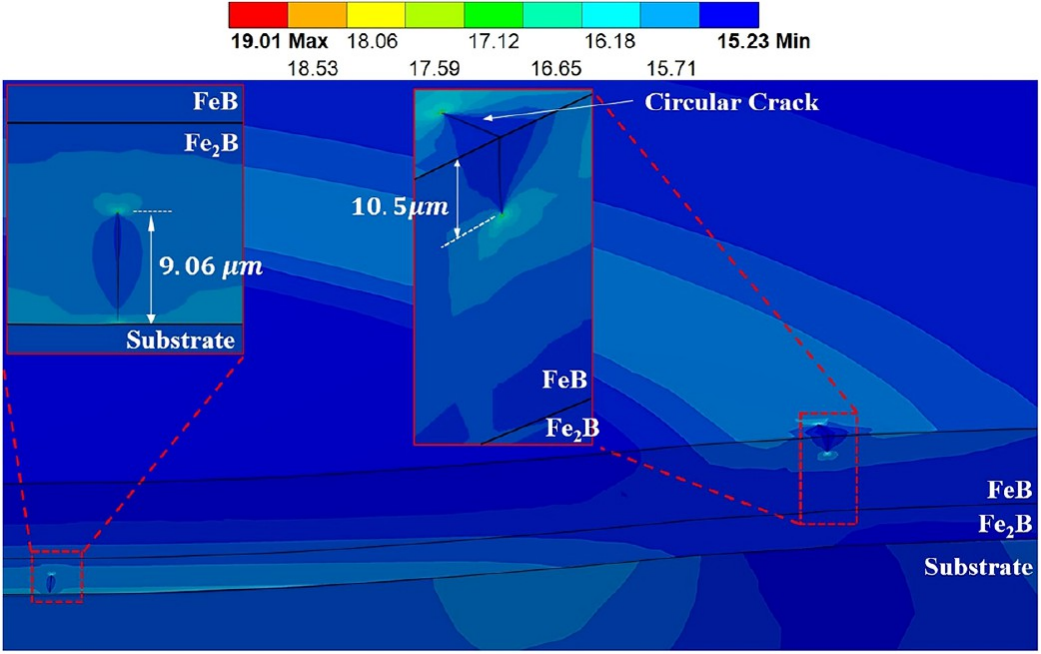
Distribution of stress intensity (MPa √m) for the growth of circular and interfacial cracks under the 6 h-600 N condition.

**Figure 14:**
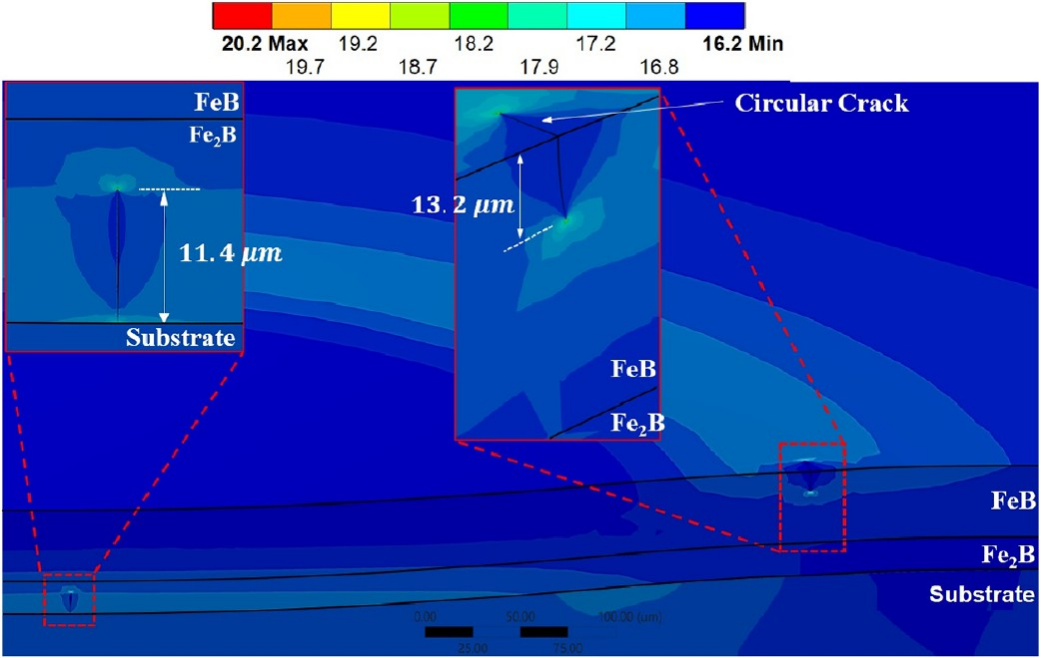
Distribution of stress intensity (MPa √m) for the growth of circular and interfacial cracks under the 6 h-650 N condition.

**Figure 15:**
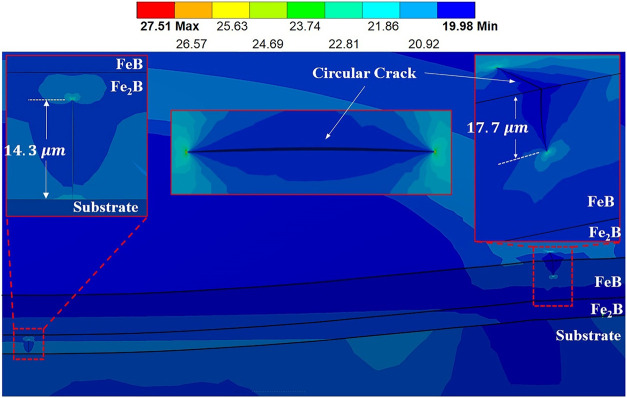
Distribution of stress intensity (MPa √*m*) for the growth of circular and interfacial cracks under the 6 h-700 N condition.

**Figure 16:**
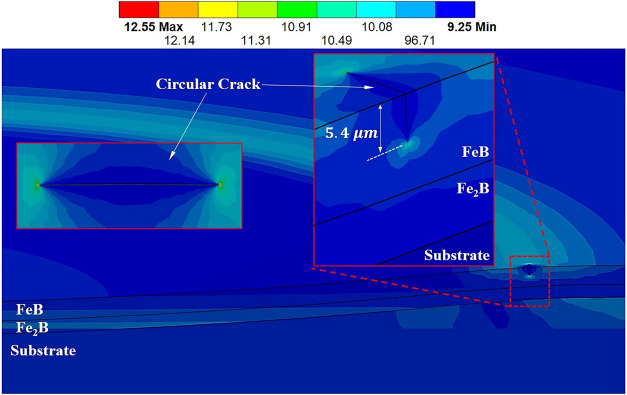
Distribution of stress intensity (MPa √m) for the growth of circular cracks under the 2 h-700 N condition.

During the unloading stage, all cases exhibit a temporary reduction in crack propagation, specifically within the 1.0–1.2 s interval, a phenomenon attributable to the elastic recovery of the substrate (*springback*). Once this relaxation period is surpassed, propagation resumes, and the maximum final crack extension is consistently reached during unloading. For the 6 h condition under 600, 650, and 700 N, the interfacial cracks initiate growth at approximately 15% of the loading stage, earlier than the surface cracks, which begin between 40–60%. However, during unloading this trend reverses, and the surface cracks reach larger extensions in all cases.

The behavior of the stress intensity factor *K*_I_ (Figure 12b) showed an evolution consistent with crack extension, increasing with the applied load and reaching its maximum values during unloading. During the loading stage, the interfacial cracks exhibited higher *K*_I_ values; however, around 80% of the cycle, this trend is reversed and the surface values become dominant. On the other hand, *springback* only affects the *K*_I_ of the surface cracks, but not that of the interfacial cracks, indicating that the stress field within the Fe_2_B phase is not influenced by this phenomenon. Under the 2 h–700 N condition, the surface *K*_I_ remained below that of the other evaluated conditions, consistent with its smaller final crack extension.

As can be observed in Figures 13–16, in all analyzed cases, the surface cracks within the FeB layer exhibit a slight inclination from their origin, which becomes more pronounced as the crack propagates toward the interior of the layer. This effect becomes evident under the 6 h–700 N condition (Figure 15), where a clear transition is observed from a perpendicular trajectory to a more inclined and wider crack opening, producing a conical geometry. Furthermore, the surface crack opening of the circular crack in this condition reaches approximately 0.7 μm, which is higher than that observed in the 2 h–700 N condition, where the opening is close to 0.2 μm, consistent with the indentation imprints obtained experimentally. This behavior agrees with the mechanisms described for cone-cracks and kinked-cone-cracks in brittle materials subjected to spherical indentation, where the stiffness contrast between the ceramic layer and the ductile substrate induces a progressive deviation of the fracture plane.^36,37^

To evaluate the sensitivity of the numerical predictions to the adopted fracture toughness values, a parametric analysis was performed, restricted to the most critical condition of the study (6 h–700 N). The fracture toughness values (*K*_IC_) of the FeB and Fe_2_B phases were varied by ±10% with respect to the baseline values taken from the literature. As summarized in Table 2, these variations produce moderate changes in the final crack propagation length (on the order of 10–15%), without modifying the propagation trajectory, the three-dimensional evolution of the crack front, or the relative severity hierarchy between surface and Fe_2_B cracks. This confirms that the conclusions of the present study are robust and governed by the interaction between the indentation-induced stress field and the relative fracture toughness of the phases, and not by a precise adjustment of the absolute fracture toughness values.

**Table 2:** Sensitivity Analysis of Crack Growth with Respect to Fracture Toughness Values for the 6 h–700 N Condition

case	*K*_IC_^FeB^ (MPa*√m)	*K*_IC_^Fe_2_B^ (MPa√m)	crack length in FeB (μm)	crack length in Fe_2_B (μm)	change in crack length vs. base (%)
Base	1.50	3.00	17.70	14.64	-
FeB −10%	1.35	3.00	20.01	14.32	+13% (surface)
FeB +10%	1.65	3.00	15.22	14.36	–11% (surface)
Fe_2_B −10%	1.50	2.70	17.75	16.03	+12% (subsurface)
Fe_2_B +10%	1.50	3.30	17.71	12.95	–10% (subsurface)
Both −10%	1.35	2.70	20.32	16.42	+15% (both)
Both +10%	1.65	3.30	15.57	12.66	–13% (both)

Additionally, in all the analyzed conditions, the position of the maximum value of the stress intensity factor *K*_I_ does not remain fixed along the crack front but instead evolves progressively during the load–unload cycle. Although the *K*_I_ curves presented in Figure 12b describe the temporal evolution of the driving force for crack propagation and allow the identification of changes in the relative dominance between surface and interfacial cracks, these curves do not provide spatial information regarding the location of the maximum *K*_I_ along the crack front. The three-dimensional analysis performed in this work makes it possible to identify that the critical point governing crack propagation is not permanently associated with the central region of the semielliptical crack front but may shift toward the ends of the front as the stress field redistributes during unloading.

This behavior is shown in Figure 17 for the most critical condition (6 h–700 N), in which a pronounced nonuniform redistribution of the stress intensity factor along the crack front is observed. In this case, the maximum value of *K*_*I*_ progressively migrates from the central region toward the ends of the crack front as the unloading stage advances, reflecting the three-dimensional evolution of the stress field and the interaction between the stiffness of the borided layer and the elastic recovery of the substrate.

**Figure 17:**
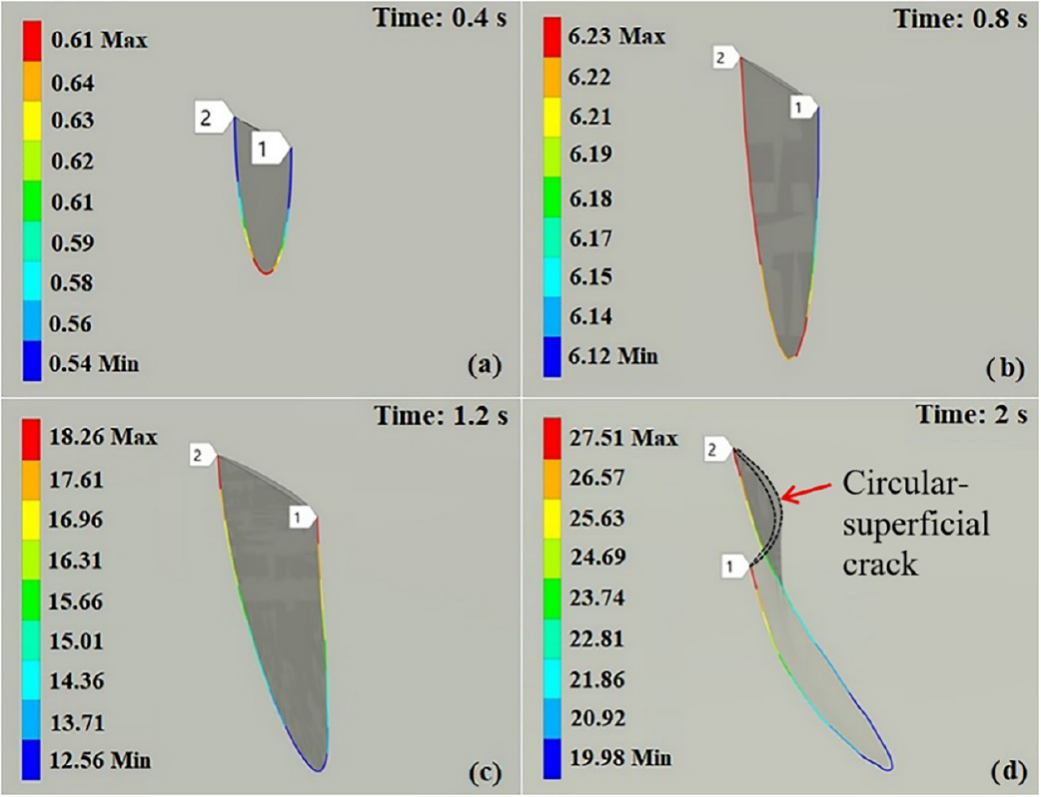
Three-dimensional evolution of the crack front and nonuniform redistribution of the stress intensity factor *K* throughout the loading–unloading cycle under the 6 h–700 N condition: (a) 40% of load step, (b) 80% of load step, (c) 20% unload step, and (d) complete unloading.

Overall, these results demonstrate that crack propagation under spherical indentation is governed not only by the magnitude of the stress intensity factor *K*_I_, but also by its spatial redistribution and by the evolution of the crack front throughout the load–unload cycle, as well as by the interaction with the elastic recovery of the substrate during unloading.

## 5 Conclusions

In this study, the mechanical response and fracture behavior of FeB/Fe_2_B layers formed on AISI 316L steel were evaluated through spherical indentation and numerical simulations, from which the following conclusions are derived.

- The powder boriding treatment produced FeB/Fe_2_B composite layers whose thickness, hardness, and elastic modulus increased with the treatment time.
- Under monotonic indentation, both systems developed circular cracks as the main cohesive damage mechanism. However, the 6 h condition exhibited cracking from 600 N and more severe surface damage, whereas the 2 h condition did not show cohesive damage until 700 N, confirming its higher fracture resistance.
- The maximum principal stresses show that the 6 h condition reaches higher magnitudes and develops a broader stress distribution, with two defined concentration zones: a surface zone and an interface one. In contrast, in the 2 h condition, the maximum stress remains confined to the surface, which significantly reduces the likelihood of interfacial damage.
- The 6h condition exhibited crack propagation in both the FeB and Fe_2_B layers, initiating at early stages of loading. In contrast, the 2h conditions showed crack growth only in the FeB layer and only at 700 N. This indicates that the greater thickness and stiffness of the 6h layer reduces its fracture resistance, whereas the thinner layer provides greater resistance to interfacial cracking.
- At the onset of unloading, crack growth is temporarily reduced by the springback of the substrate in both systems. After the elastic recovery, the adhesion between the layer and the substrate maintains the coating in a bent state, reinitiating crack propagation. These observations demonstrate the influence of the elastoplastic behavior of the substrate on the fracture behavior of the layers.
- During the initial portion of the loading stage, the crack in Fe_2_B initiates earlier than in FeB in all cases, due to the higher stress concentration at the Fe_2_B/substrate interface. However, toward the end of the loading stage this trend reverses, and the surface cracks become dominant. This occurs because, as the interfacial crack extends, it approaches the compressive stress field beneath the indenter–FeB layer contact zone, thereby limiting its propagation.
